# UPR-Mediated Membrane Biogenesis in B Cells

**DOI:** 10.1155/2012/738471

**Published:** 2011-11-01

**Authors:** Joseph W. Brewer, Suzanne Jackowski

**Affiliations:** ^1^Department of Microbiology and Immunology, College of Medicine, University of South Alabama, Mobile, AL 36688, USA; ^2^Department of Infectious Diseases, St. Jude Children's Research Hospital, Memphis, TN 38105, USA

## Abstract

The unfolded protein response (UPR) can coordinate the regulation of gene transcription and protein translation to balance the load of client proteins with the protein folding and degradative capacities of the ER. Increasing evidence also implicates the UPR in the regulation of lipid synthesis and membrane biogenesis. The differentiation of B lymphocytes into antibody-secreting cells is marked by significant expansion of the ER, the site for antibody synthesis and assembly. In activated B cells, the demand for membrane protein and lipid components leads to activation of the UPR transcriptional activator XBP1(S) which, in turn, initiates a cascade of biochemical events that enhance supplies of phospholipid precursors and build machinery for the synthesis, maturation, and transport of secretory proteins. The alterations in lipid metabolism that occur during this developmental transition and the impact of membrane phospholipid restriction on B cell secretory characteristics are discussed in this paper.

## 1. Introduction

Activated B lymphocytes proliferate and proceed along distinct developmental pathways that determine their function and fate. Specifically, responding B cells can rapidly differentiate in extrafollicular sites into short-lived antibody-secreting cells that predominantly secrete IgM antibodies [1]. Alternatively, responding B cells can enter germinal centers, undergo somatic hypermutation and isotype switching, and then become memory B cells or long-lived antibody-secreting cells [2]. Extrinsic factors, including the nature of the antigen and T cell help in the form of membrane-bound molecules and soluble cytokines, play key roles in regulating B cell responses. However, intrinsic signals are also pivotal in directing the fate of responding B cells as evidenced by the critical role of the unfolded protein response (UPR) transcription factor XBP1(S) in driving the differentiation of antibody-secreting cells [3, 4], the effectors of humoral immunity. Here, we discuss the current understanding of the relationship between the UPR, lipid biosynthesis and organelle biogenesis in activated B cells. 

## 2. Lipid Supply and Demand

B lymphocytes proliferate and differentiate into antibody-secreting cells upon interaction with specific antigen or certain Toll-like receptor (TLR) ligands. When B cells are stimulated to enter the cell cycle and proliferate, the mechanisms that control the membrane phospholipid supply in rapidly dividing cells are engaged. The division of one cell into two daughter cells requires a doubling of membrane content during cell cycle progression [5]. Phosphatidylcholine (PtdCho) is the major membrane phospholipid in mammalian cells and is a precursor to the two other most abundant membrane phospholipids, sphingomyelin (SM) [6] and phosphatidylethanolamine (PtdEtn) [7]. PtdCho and the other phospholipids accumulate in a periodic manner during S phase, coincident with DNA synthesis. The net increase in membrane PtdCho results from an interaction between cell cycle-dependent oscillations in the rates of PtdCho biosynthesis and degradation. PtdCho synthesis is stimulated very early during G1 phase [8–10], but is accompanied by rapid PtdCho turnover. Two phospholipases have been implicated in the PtdCho turnover associated with cell cycle progression, the group VIA calcium-independent phospholipase A_2_ [11] and the neuropathy target esterase [12]. Near the G1/S transition, PtdCho turnover is diminished substantially, yielding a net increase in membrane PtdCho. Toward the latter part of the cell cycle, prior to cytokinesis, PtdCho synthesis is downregulated [5]. This cyclic variation in the supply of membrane phospholipid for cell proliferation is maintained in the absence of differentiation.

B cells are unique, however, and in addition to proliferation also undergo a subcellular membrane expansion as they differentiate into antibody-secreting cells after stimulation. There is a major increase in synthesis and secretion of immunoglobulin (Ig) heavy (H) and light (L) chains [13]. Nascent Ig chains are cotranslationally translocated into the endoplasmic reticulum (ER), an oxidizing, calcium-rich environment containing many resident molecular chaperones and folding enzymes [14]. Within this specialized protein folding compartment, H and L chains are assembled into functional antibodies. Induction of high-rate Ig synthesis during the differentiation process is accompanied by expansion of the rough ER membrane, at least 3- to 4-fold in surface area and volume [15, 16]. Thus, both proliferation and differentiation require an increased supply of phospholipids to fuel membrane and organelle biogenesis. To meet this demand, the synthesis of phospholipids, particularly PtdCho, increases when B cells are activated [15, 17]. 

## 3. Phosphatidylcholine Synthesis

**Figure 1:**
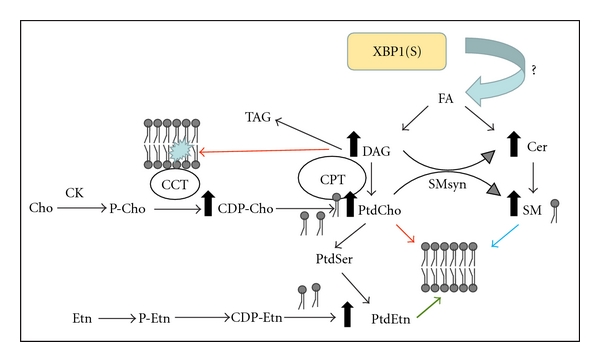
Activation of membrane phospholipid synthesis. Expression of XBP1(S) stimulates *de novo* fatty acid (FA) synthesis and the new FAs are incorporated into diacylglycerol (DAG) and ceramide (Cer), immediate precursors of phosphatidylcholine (PtdCho), and sphingomyelin (SM) phospholipids, respectively. The mechanism of stimulation by XBP1(S) has not yet been defined. Elevation of the DAG level alters the membrane lipid composition which leads to activation of the choline cytidylyltransferase (CCT) enzymes which produce CDP-choline (CDP-Cho). The DAG and CDP-Cho precursors are converted to PtdCho by the choline phosphotransferase (CPT) enzymes. Excess DAG which is not incorporated into phospholipid, is redirected and incorporated into triacylglycerol (TAG) which can accumulate in lipid droplets. PtdCho conversion to SM is mediated by sphingomyelin synthase (SMsyn). PtdCho conversion to phosphatidylethanolamine (PtdEtn) is routed through phosphatidylserine (PtdSer). PtdEtn can also be synthesized from ethanolamine (Etn) and DAG by the alternative CDP-ethanolamine (CDP-Etn) pathway. Elevation of all three phospholipids, PtdCho, SM, and PtdEtn, contributes to membrane biogenesis during B cell activation. Cho, choline; P-Cho, phosphocholine; CK, choline kinase; Etn, ethanolamine; P-Etn, phosphoethanolamine.

The predominant means for PtdCho biosynthesis in mammalian cells proceeds via the three steps of the cytidine diphosphocholine (CDP-choline) pathway [18] (Figure 1). First, choline kinase (CK) phosphorylates choline in the presence of ATP to yield phosphocholine. CK*α* and CK*β* are two isoforms which are soluble proteins found in the cytosol [19, 20]. Second, choline cytidylyltransferase (CCT) converts phosphocholine to CDP-choline in the presence of CTP, and this is the rate-limiting step in the pathway [21]. In every cell type examined thus far, including B cells [17], CCT catalyzes the slow step in the pathway and thereby determines the rate of PtdCho formation. Comparatively small amounts of CDP-choline are found in cells, in relation to other phospholipid precursors, as CDP-choline is utilized almost immediately after it is made. CCT, including all mammalian isoforms, transiently associates with the ER membrane and the lipid composition of the ER membrane governs CCT association and activity [22]. Elevated expression of CCT stimulates PtdCho synthesis but often does not result in an increased amount of cellular PtdCho in most proliferating cells due to compensatory elevation of PtdCho turnover mediated by phospholipases [23, 24]. Third, the phosphocholine moiety of CDP-choline is transferred to diacylglycerol (DAG), producing PtdCho. This final step can be catalyzed by either cholinephosphotransferase (CPT1) or choline/ethanolaminephosphotransferase (CEPT1), a bifunctional enzyme that can synthesize both choline- and ethanolamine-containing phospholipids. The CPT enzymes are integral membrane proteins, and the CPT1 is found with the Golgi apparatus while the CEPT1 associates with the ER [25, 26]. Here, we refer to the activities of CPT1 [27] and CEPT1 [28] collectively as CPT activity. The locations of the CPT enzymes designate the subcellular sites of membrane biogenesis; however, enforced overexpression of CPT activity does not enhance PtdCho synthesis [29, 30]. Rather, the supply of CDP-choline and DAG determine the amount of PtdCho. Thus, elevated expression of the CPT enzymes can be considered as a marker for Golgi and/or ER membrane expansion, but not necessarily as a driver of membrane phospholipid synthesis. 

In lipopolysaccharide- (LPS-) stimulated splenic B cells, CK activity remains fairly constant, CCT activity modestly increases *≈*2-fold, and CPT activity increases *≈*6-fold [15]. These modulations of the CDP-choline pathway enzymes in LPS-stimulated splenic B cells correlate with a 6- to 7-fold increase in PtdCho synthesis [15, 31]. Our studies using the CH12 B cell lymphoma indicate that increased CCT activity is pivotal for enhanced flux through the CDP-choline pathway in LPS-stimulated B cells [17]. In this system, the CCT expression and enzyme specific activity do not increase when assayed under optimal *in vitro* conditions following LPS stimulation. However, radiolabeling experiments of stimulated cells demonstrate that the formation of CDP-choline is substantially enhanced, indicating allosteric activation of CCT by membrane lipids. Indeed, microsomal lipids isolated from stimulated cells contain an elevated amount of DAG and significantly stimulate the activity of purified recombinant CCT, compared to lipids isolated from unstimulated cells. Thus, in this case, the formation of DAG is key to stimulation of PtdCho synthesis: first, by activating CCT, and second, by providing substrate for the CPT enzymes. The CCT, in turn, governs the fate of the DAG as DAG is incorporated either into phospholipid under permissive CCT conditions or into triacylglycerol (TAG) when the CCT activity is reduced [32] (Figure 1). 

## 4. A “Physiologic” UPR

ER stress occurs when the load of client proteins exceeds the folding capacity of the ER, a condition that can be catastrophic if unresolved. To rebalance load with capacity in the ER, thereby relieving ER stress, the UPR can slow the flow of nascent polypeptides into the ER lumen and enhance the ER machinery needed for folding and/or disposal of client proteins [33, 34]. The mammalian UPR is orchestrated by a trio of signaling pathways that are separately initiated by three ubiquitously expressed ER transmembrane proteins: PERK (PKR-like ER kinase) [35, 36], ATF6 (activating transcription factor 6) *α* and *β* [37, 38], and IRE1 (first identified in a yeast mutant with inositol requiring phenotype) *α* and *β* [39, 40]. The activation status and role of each UPR pathway has been examined during the differentiation of antibody-secreting B cells. 

The PERK protein possesses a serine/threonine kinase domain in its cytoplasmic region through which it mediates translational attenuation [35, 36]. Upon activation, PERK phosphorylates the *α* subunit of eIF-2 (eukaryotic initiation factor-2) on serine 51, thereby impeding formation of translation initiation complexes and slowing the flow of nascent polypeptides into the ER [41, 42]. PERK does not appear to be activated during the differentiation of antibody-secreting B cells [43, 44]. In support of this concept, studies of gene-targeted mice reveal that the PERK pathway is dispensable for antibody secretion [43]. 

ATF6*α* and ATF6*β* are type II ER transmembrane proteins [37, 38]. Upon UPR activation, ATF6 traffics from the ER to the Golgi complex where it is clipped by the Site-1 and Site-2 proteases [45, 46]. Once liberated from the membrane by this process of intramembrane proteolysis, the cytosolic N-terminal domain of ATF6 moves into the nucleus where it functions as a transcriptional activator of genes encoding ER resident molecular chaperones, folding enzymes and components involved in ER-associated degradation (ERAD) of misfolded proteins [37, 38, 47–49]. While ATF6*α* and *β* are both functional, only ATF6*α* appears essential for induction of ER stress responsive genes and survival of cells subjected to ER stress conditions [48, 49]. Overexpression of active ATF6*α* is sufficient to drive synthesis of fatty acids and phospholipids and to induce expansion of rough ER [50], suggesting that this UPR pathway might participate in the differentiation of antibody-secreting B cells. Indeed, ATF6*α* is activated in LPS-stimulated B cells [43, 51, 52]. However, recent studies of ATF6*α*-deficient mice indicate that ATF6*α*, like PERK, is dispensable for the differentiation of antibody-secreting B cells (Brewer et al., manuscript in preparation). 

The IRE1 proteins contain a serine-threonine kinase module and a C-terminal endoribonuclease domain in their cytoplasmic regions [39, 40]. Upon activation, IRE1 executes site-specific cleavage of *Xbp1* (X-box binding protein 1) mRNA. A 26-nt intron is excised and an undefined mechanism then ligates the resulting 5′ and 3′ fragments, yielding a spliced *Xbp1* mRNA with an altered reading frame [53–55]. Both unspliced and UPR-spliced *Xbp1* transcripts encode basic leucine zipper (bZIP) transcription factors, XBP1(U) and XBP1(S), respectively. The XBP1(S) factor exhibits enhanced transactivating capacity and greater stability as compared to XBP1(U) [53–56]. Like ATF6*α*, XBP1(S) is sufficient to upregulate synthesis of fatty acids and phospholipids and to drive expansion of rough ER [30, 50]. *Xbp1* is essential for optimal induction of genes encoding proteins that function throughout the secretory pathway and for proper development of the ER in a variety of specialized secretory cell types [57, 58]. When B cells are stimulated to secrete antibody, *Xbp1* mRNA increases and undergoes UPR-mediated splicing to yield XBP1(S) [3, 52, 53], a factor required for the generation of antibody-secreting B cells [3, 4]. Thus, the physiologic UPR of activated B cells features the IRE1/XBP1 pathway. 

## 5. XBP1(S), Lipid Synthesis, and ER Biogenesis

*Xbp1* is required for embryonic development [59]; thus, the role of this UPR transcription factor in lymphocytes was first investigated using the *Rag-2* complementation system [4]. Those experiments revealed that XBP1-deficient B cells are markedly defective in antibody secretion *in vivo* in response to immunization and *in vitro* in response to LPS. Importantly, it was shown that XBP1(S), but not XBP1(U), effectively restores the ability of XBP1-deficient B cells to secrete antibody in response to LPS *in vitro* [3] and is sufficient to drive ER expansion [30, 58]. More recently, the Cre-*loxP* system has been employed for selective deletion of *Xbp1* in B cells and studies using this system have corroborated the earlier findings [60, 61]. Using this system, the abundance of PtdCho was shown to increase in LPS-stimulated XBP1-deficient B cells, but to a lesser degree than in wild-type cells [62]. The levels of PtdCho, SM, and phosphatidylinositol were significantly reduced in activated XBP1-deficient B cells, but PtdEtn, phosphatidylserine, and phosphatidylglycerol were similar to corresponding amounts in wild-type activated B cells. In addition, a meager, but discernible, expansion of the rough ER was observed in LPS-stimulated XBP1-deficient B cells [62].

PtdCho is most drastically affected by XBP1 deficiency because it is the most abundant phospholipid of the ER membranes. SM is derived directly from PtdCho, where the phosphocholine headgroup of PtdCho is transferred to ceramide by the SM synthase [63] (Figure 1). Thus, a reduction in PtdCho availability would be reflected by a reduction in SM. The pathway for PtdCho conversion to PtdEtn is not as direct, however, and a second pathway of PtdEtn synthesis via CDP-ethanolamine can bypass a deficiency in PtdCho [64]. Thus, the amount of PtdEtn is less affected following activation of XBP1-deficient B cells and PtdEtn increases to almost the same extent as in activated wild-type B cells. On the other hand, the enforced expression of XBP1(S) in NIH-3T3 fibroblasts leads to a substantial increase in PtdEtn [30], augmenting the XBP1(S)-independent mechanism(s) of lipogenesis. The *de novo* synthesis of ceramides, key precursors in SM production, is upregulated upon LPS stimulation [65] and contributes to the increase in SM. Inhibition of ceramide formation impairs ER expansion and protein glycosylation in the ER lumen [65], suggesting a link among these processes. These data establish that XBP1 is required for maximal increases in PtdCho, SM, and rough ER in LPS-stimulated B cells, but the mechanisms by which XBP1 mediates these events remain to be elucidated. The scheme in Figure 1 shows a cascade of biochemical events which illustrates how XBP1(S) stimulation of fatty acid synthesis [50] is a key feature that drives membrane phospholipid expansion in B cells [17]. Furthermore, these data suggest that XBP1-independent mechanisms, as yet undefined, must also contribute to the regulation of PtdCho synthesis and ER biogenesis during the differentiation process.

It has been proposed that the escalation of Ig synthesis in differentiating B cells taxes the protein folding machinery of the ER and, consequently, triggers the UPR [3]. This model was supported by an experiment showing reduced induction of XBP1(S) in B cells that had undergone *ex vivo* Cre-mediated deletion of IgH chain prior to LPS stimulation [3]. In contrast, recent studies have shown strong induction of XBP1(S) in *μ*_s_^−/−^ B cells stimulated with LPS [60, 62], indicating that increased synthesis of soluble *μ* H chains is not a prerequisite for UPR activation. In keeping with these data, we previously showed that synthesis of XBP1(S) precedes induction of maximal Ig translation in LPS-stimulated CH12 B cells [52], indicating that the IRE1/XBP1 pathway is activated at an earlier stage of the differentiation process. What then is the signal(s) for UPR activation in stimulated B cells? This remains a fundamental question, and its answer is integral to understanding the mechanisms that drive development of antibody-secreting B cells.

## 6. Phosphatidylcholine Synthesis and UPR Signaling

Mammals express three CCT isoforms that are similar in enzymatic activity and regulation. CCT*α* is encoded by the *Pcyt1a* gene whereas CCT*β*2 and CCT*β*3 are encoded by alternatively spliced transcripts from the *Pcyt1b* gene [66]. CCT*α* is predominantly expressed in most tissues, including B cells [17], and is required for early embryonic development [67]. Tissue-specific deletion of the *Pcyt1a* gene using the Cre-*loxP* system has revealed critical roles for CCT*α* in specialized secretory cells, including surfactant lipid production and secretion by alveolar epithelial cells [68], assembly and secretion of lipoproteins by hepatocytes [69], and cytokine secretion by activated macrophages [70]. We recently showed that selective deletion of CCT*α* significantly hampers the ability of B cells to upregulate PtdCho synthesis upon stimulation, and interestingly, this correlates with heightened induction of the IRE1/XBP1 branch of the UPR [31]. 

**Figure 2:**
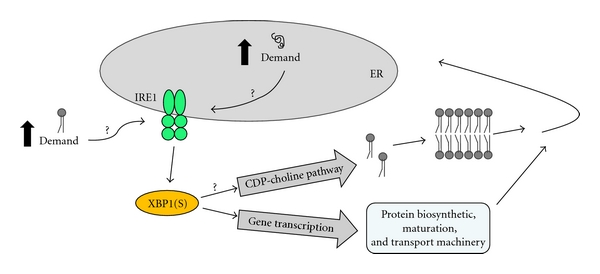
XBP1(S), lipids, and secretory pathway machinery in ER biogenesis. In activated B cells, we propose that increased demand for lipids as well as increased demand on the protein folding capacity of the ER promotes induction of the XBP1(S) transcriptional activator via the IRE1/XBP1 branch of the UPR. The means by which these demands are sensed by the IRE1/XBP1 pathway remain unclear. XBP1(S), via transcriptional control, upregulates expression of a large cohort of proteins involved in the synthesis, maturation, and transport of cargo proteins within the secretory pathway. Much of this secretory machinery localizes to the ER. XBP1(S), via mechanisms that are poorly understood, also drives lipid biosynthesis, including production of the major phospholipid PtdCho by the CDP-choline pathway. Thus, XBP1(S) coordinates mechanisms that supply both the lipid and protein components necessary for construction of the ER.

When challenged with a T cell-dependent protein antigen, the animals harboring CCT*α*-deficient B cells were unable to produce normal levels of IgG but secreted hyperlevels of IgM [31]. The correlation between the reduced PtdCho synthesis and elevated IgM secretion in the CCT*α*-deficient B cells was counterintuitive, however, based on the implied need for membrane PtdCho expansion during plasma cell differentiation. Investigation of the UPR components revealed that the impaired production of PtdCho triggers IRE-mediated splicing of *Xbp1* mRNA early after activation, thereby promoting differentiation of IgM-secreting cells. The inability of CCT*α*-deficient B cells to undergo isotype switching correlates with a proliferation defect. However, blocking proliferation by a different mechanism did not elicit XBP1(S) activation, supporting the idea that the early and potent induction of XBP1(S) by PtdCho deficiency in CCT*α*-deficient B cells accelerates and augments the transition into antibody secretion. From these observations, we propose that the IRE1/XBP1 branch of the UPR responds to increased demand for phospholipids as well as increased demand on the protein folding capacity of the ER (Figure 2). In agreement, restriction of either PtdCho [71] or fatty acid synthesis [72] has been shown to elicit activation of UPR components in other systems. It is intriguing to speculate that lipid supply might function as a metabolic cue for induction of the IRE1/XBP1 pathway in activated B cells.
